# Provision of dental services for vulnerable groups: a scoping review on children with special health care needs

**DOI:** 10.1186/s12913-021-07293-4

**Published:** 2021-12-04

**Authors:** Peivand Bastani, Mohammadtaghi Mohammadpour, Arash Ghanbarzadegan, Giampiero Rossi-Fedele, Marco A. Peres

**Affiliations:** 1 Health Human Resources Research Center, School of Management and Medical Informatics, Shiraz University of Medical Sciences, Ghasrdasht street, Shiraz, Iran; 2 Health Human Resources Research Center, School of Management and Medical Informatics, Shiraz University of Medical Sciences, Shiraz, Iran; 3 Australian Research Centre for Population Oral Health (ARCPOH), Adelaide Dental School, University of Adelaide, Adelaide, South Australia Australia; 4 Faculty of Health and Medical Sciences, Adelaide Dental School, University of Adelaide, Adelaide, South Australia Australia; 5 National Dental Research Institute, Singapore, 5 Second Hospital Ave, Singapore, 168938 Singapore

**Keywords:** Dentistry service, Provision, Children with special health care needs, Dental health, Oral health, Disparity

## Abstract

**Background:**

The provision of dental services for children with special health care needs (CSHCN) needs to be considered by policymakers. This study is aimed to explore the determinant factors affecting dental and oral services provision for this vulnerable group.

**Methods:**

A review was conducted applying the 9-steps approach. Five scientific databases of PUBMED, SCOPUS, Web of Science and PROQUEST and EMBASE were searched up to 10.07.2021, applying appropriate keywords. Thematic analysis was used to analyse the extracted data, and a conceptual map was developed according to JBI manual for evidence synthesis.

**Results:**

From the abstracts of the 136 articles that fulfilled the inclusion criteria, 56 articles were included. Five main themes were identified as determinants affecting the provision of dentistry services for CSHCN, including needs assessment, policy advice, oral health interventions, providers’ perception and access barriers. According to the developed conceptual map, assessing the needs of CSHCN can lead to particular policy advice. Regarding the policies, appropriate oral health interventions can be presented. These interventions, along with providers’ perception about service delivery to CSHCN and the barriers to access them, determine the provision of dentistry services for CSHCN.

**Conclusions:**

An effective needs assessment of CSHCN and their parents/carers can lead to evidence-informed policymaking and applicable policy advice according to the needs. Then policymakers should develop interventions to improve the community’s health literacy, as well as support the seeking behaviours for appropriate services. Policymakers should also consider how to limit the barriers to accessing oral and dental health by CSHCN to decrease disparities.

**Supplementary Information:**

The online version contains supplementary material available at 10.1186/s12913-021-07293-4.

## Introduction

People with special health care needs (SHCN) are defined as those having long-term physical, behavioural, emotional and developmental conditions that require attention and health care [1]. Different causes have been identified, such as congenital, developmental, traumatic or environmental associated reasons for these conditions, which lead to a limitation in daily activities [2]. These conditions affect a wide range of the world’s population at all ages and social classes, but its prevalence varies from region to region. Based on the World Health Organization’s report on disability, the estimated population of Children with Special Health Care Needs (CSHCN) among 14-year-old and younger individuals with “moderate or severe disability” and “severe disability” were 93 million and 13 million, respectively as the prevalence of disability for them were 5.1 and 0.7% in 2004 [3]. This prevalence was reported by the U.S. Department of Health and Human Services in 2009–2010. According to this report, 15.1% of American children (about 11.2 million children) had special health care needs [4]. This can directly cause a financial burden for those families with CSHCN because of their healthcare expenditures [5].

People with special needs are considered as high-risk and vulnerable groups, not only because of the problems caused by disability but also because of the limitations that society imposes on them. In some societies, people with special needs are usually kept out of society and have limited access to educational and health facilities [6]. These conditions directly affect general health and, consequently their oral health [7, 8]. In general, CSHCN is at higher risk for health problems. They have less access to oral health services, and their underlying conditions can affect their oral health status [9]. Many of them have sensory and motor disabilities, which makes attending a routine dental appointment difficult [10, 11]. It was reported that 62.5% of the children’s parents/carers admitted that their children had difficulty brushing their teeth [12]. Also, the parents/carers of these children often delay dental treatment due to anxiety [13]. On the other hand, dentists are less likely to treat people with special needs for various reasons [14]. Numerous other factors, such as the economic status, parental information levels, drug therapies, and systemic conditions, are related to the oral health status of these children [15–18].

While many potential barriers are reported for the provision of dental services to the general population, these issues become more considerable for CSHCN. For instance, the nonavailability of dentists is reported as a major barrier to access dental care services [19]. Other barriers of providing dental services include lack of appropriate government policies and dental benefit schemes [20]. These barriers can be intensified for people with any kind of disability, particularly children. Some of these barriers are related to physical, structural, geographical, professional, or behavioural determinants that can simply restrict their access to the oral and dental health services [21], and the others are related to the governments and health policymakers to provide more comprehensive and accessible packages for them.

More than what was stated, it is important to notice that according to the reports, the prevalence of caries in CSHCN is higher than in other children [9]. The most common oral diseases in this group are the higher prevalence of dental caries and periodontal disease [22, 23]. Considering all the above, the issue becomes more highlighted, and various ways are applied to reduce the problems of people with special needs to receive dental services such as creating ramps for the disabled, proper restroom, dental chairs suitable for wheelchairs, increasing various courses for parents/carers and dental students, etc. All these interventions greatly depend on the provision of the appropriate services according to CSHCN` needs. In this regard, many universities also offer courses for undergraduate students to increase the willingness of graduate dentists to treat people with special health needs by treating in their clinical hours [24, 25].

Therefore, according to evidence and to the best of our knowledge, it is obvious that CSHCN as a vulnerable group requires to be mentioned from all the aspects of their health as well as dental and oral health and providing dental services along with preventive oral interventions should be considered for them, but the question is that what are the main determinant factors affecting on dental and oral services provision in children with special health care needs.

In order to answer this question and because of the lack of a comprehensive systematic framework for updated studies in this area, this scoping review aimed to review and update the literature and develop the conceptual boundaries of the topic for the provision of dental and oral services in children with special health care needs.

## Method

Scoping reviews are generally conducted to indicate the nature and extent of the research evidence [26]. The present scoping review has adopted the 9-step approach proposed by Peters et al. (2015) according to the JBI manual of evidence synthesis [27].

**Fig. 1:**
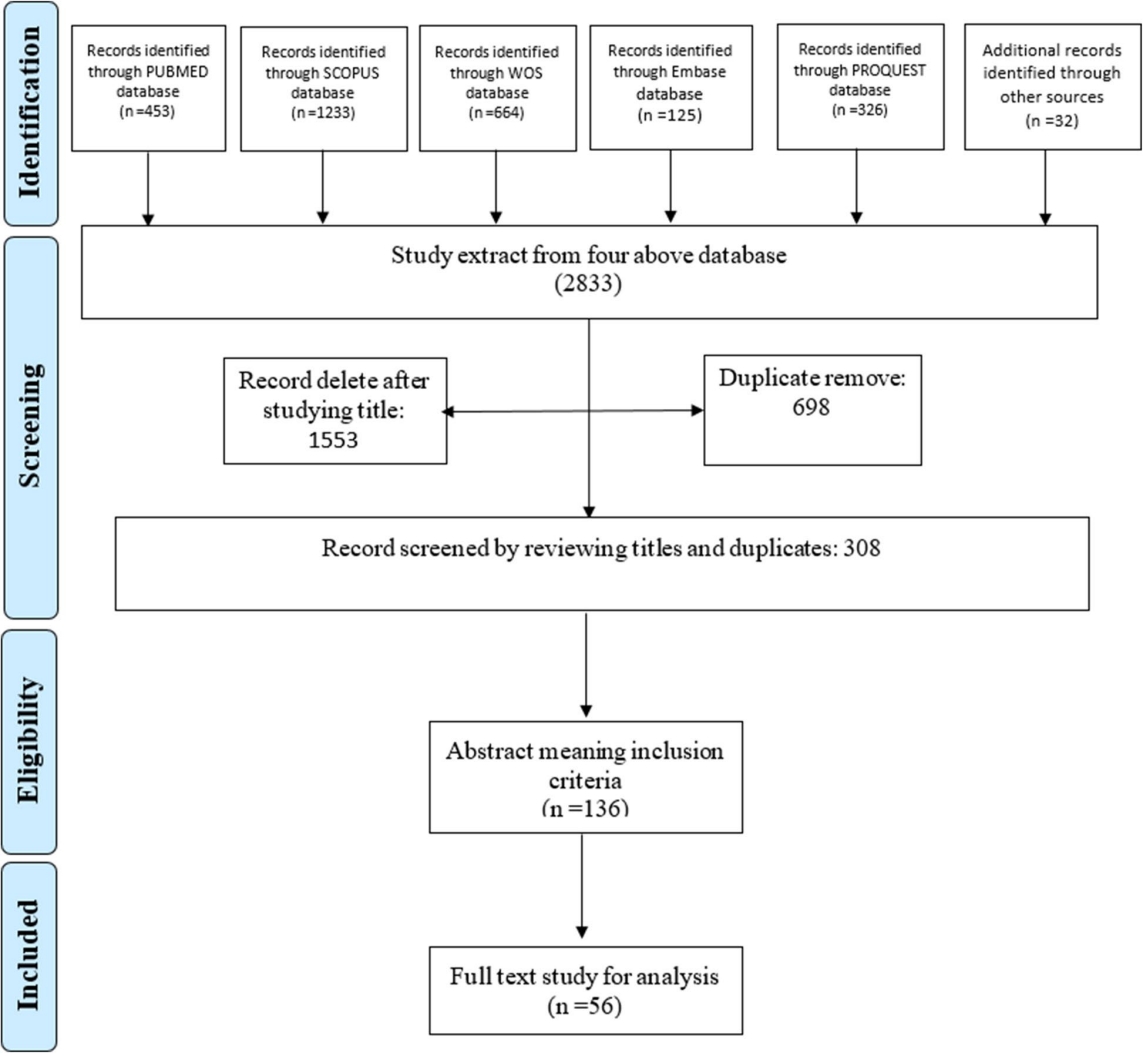
PRISMA flowchart of the scoping review

I. ***Defining and arranging the objective and question of the scoping review***
  At the first step, the research team defined the objective of the review, which was achieving a conceptual map for dental services provision of CSHCN. With this purpose, the review question was aligned as follows: “what are the main determinants of dental services’ provision in CSHCN”?
II. ***Developing and aligning the inclusion criteria with the objective and question***
  First, the PCC that stands for participants, concept and context was developed as follows: the population is defined as the dental services provided for CSHCN, the concept includes the provision of dental services in CSHCN. Context also includes the social, cultural, political, financial and technological aspects that surround the content. According to this, the inclusion criteria were all the articles having the English full-text in any qualitative or quantitative designs, mix-methods, commentaries, viewpoints and editorials with the aim of dentistry services provision for CSHCN. The conference proceedings, policy papers, reviews, guidelines and instructions were excluded.
III. ***Describing the planned approach to evidence searching and selecting***
  In this step, the search strategy was designed applying the related keywords the same as dental health, oral health, dentistry, oral hygiene, special health care needs, disability, special needs, unmet needs, child and children and other synonymous words or phrases. To achieve more sensitivity, the Boolean operator OR was used between all the synonyms, and the operator AND was applied to combine them. The search limitations were the English language and the time limit between 01.01.2000, up to 10.07.2021. Table A-Supplement reports the search strategy syntax.
IV. ***Searching for the evidence***
  Five scientific databases of PUBMED, SCOPUS, ISI Web of Science and PROQUEST and EMBASE were searched systematically, applying the search strategy’s keywords (Table A-Supplement). Google Scholar was also searched at the last stage for searching according to the titles.
V. ***Selecting the evidence***
  The following systematic search was conducted step by step according to the PRISMA [28] flowchart for clarifying the results from the search of the databases, removing the duplications, reviewing the articles via their titles and abstracts and selecting the related articles according to the objective of the review, retrieving the full texts and finally going through the reference lists for a hand-search through Google Scholar. The PRISMA flowchart shows the details in Fig. 1.
VI. ***Extracting the evidence***
  A data extraction form was designed according to the study’s objective. This form contains some information such as the name of the author, the year of publishing the study, study place, aim of the study, study design, study setting and the main results as the determinants of the provision of dental services in CSHCN.
VII. ***Charting the evidence***
  In this step, the data is charted and summarized descriptively and with the logic of achieving the objective of the study. This chart distinctly shows the characteristics of the included studies according to the data of the extraction form (Table B-Supplement).

### Summarizing the evidence in accordance with the objective and question.

In order to categorize and summarize the data, the thematic analysis was used via six following steps: First, the extracted data from the full-text of the articles were reviewed several times and compared with the original text, then the coding process was started, and the appropriate labels were assigned to the initial codes, in another word, in an open coding process, the meaningful units of the extracted data which better answer to the review’s research question were highlighted and labeled as the initial codes. Then, in the third step, the initial codes were reviewed and integrated to achieve the final codes. The finalized codes became integrated, refined and categorized one more time, to reach the sub-themes according to the study’s objective. In the fifth step, the main themes were appeared by categorizing the sub-themes according to their main concepts, these main themes finally were reviewed and labelled, and at the last step, the main themes and sub-themes were tabulated for better illustration and comprehension. MAXQDA software version 10 was used to analyze the data.

VIII. ***Consultation of experts***
  This step (consultation of experts) is recommended throughout the scoping review according to the JBI manual for evidence synthesis [27]. As the final aim of the scoping review is to develop the conceptual boundaries of the topic and achieve a final map to show the graphic logical relationships between the main themes and sub-themes. In this step, a thematic network was illustrated applying VISIO plan software, and the final map was consulted with 3 of the experts in the scope of health policymaking and oral health. These three experts have a related academic education as well as scientific reputation in the area of oral and dental health policymaking. For this purpose, after.
  finalyzing the “data charting” and tabulating the results of main and sub-themes, a virtual mini-expert panel was conducted with three aforementioned experts and two of the researchers who are experts in the area of oral and dental health policymaking. The main results were presented and discussed via the virtual panel, and the viewpoints of the experts were achieved to become sure of the comprehensiveness and robustness of the results and the appropriatness of the suggested map.

## Results

**Fig. 2:**
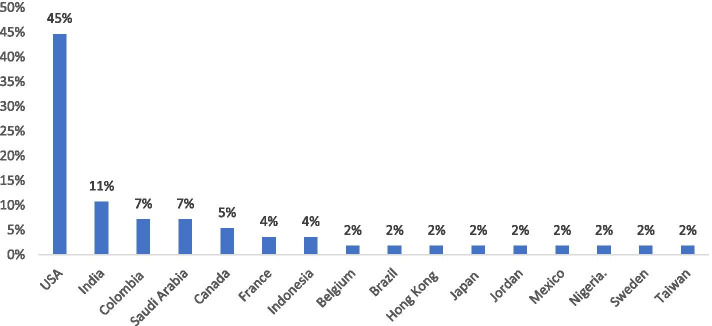
Distribution of the included articles according to their place

Results show that among 136 articles’ abstracts that fulfilled the inclusion criteria, 56 full texts were included to be analysed (Fig. 1). Table B-Supplement shows the characteristics of these articles. According to Fig. 2, most of the included articles were conducted in the USA (45%).

**Fig. 3:**
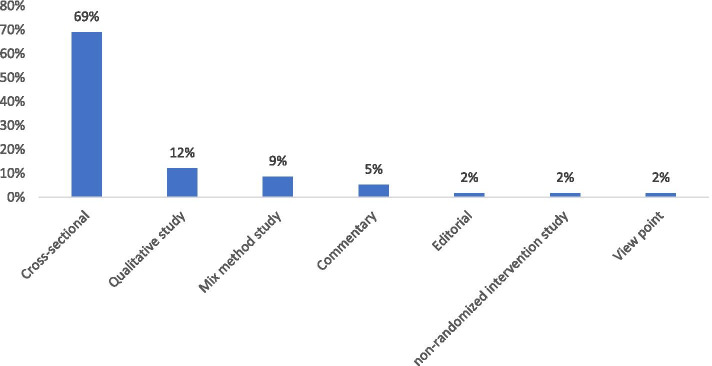
Distribution of the included articles according to their study type

Considering the type of study, Fig. 3 shows that most of the included articles have a cross-sectional design (69%), qualitative studies were in the second order (12%).

**Fig. 4:**
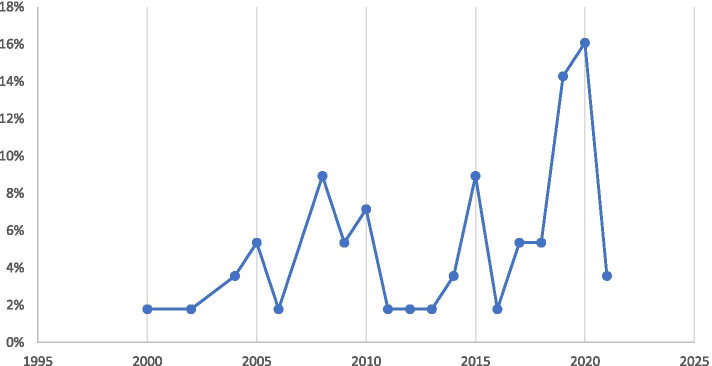
Distribution of the included articles according to the year of publication

Finally, other descriptive results demonstrated that most of the articles were published in 2020 that can indicate the highlighted significance of the issue among the researchers (Fig. 4).

The thematic analysis of the data has summarized in Table 1. Five main themes and 47 related sub-themes are created according to Table 1. The main themes include providers` perception, need assessment, access barriers, policy advice and interventions for oral health improvement, determining the main dimensions of the dental and oral services` provision that are needed to be considered. More descriptions of each of these dimensions are as follows:

**Table 1:** Dimensions to be considered for children with special needs` dentistry service

Main dimensions	Sub-dimensions	References
**Need assessment**	Caries-risk assessment	[29]
	Improving the health literacy (children, parents and caregivers)	[29–33]
	Quality of care of caregivers and caregivers burden	[34, 35]
	Poor oral hygiene status	[9]
	Utilize preventive dental care	[36, 37]
	Dentist visit/Timely referral to the dentists	[35, 37–39]
	Difficulty in performing proper tooth brushing	[31]
	Level of intellectual disability	[8]
**Providers perception**	Dentists` feelings and perceptions	[40]
	Paediatricians`/dentists` knowledge and experience	[35, 39, 41, 42]
	Dentists` specialized training like empathy training	[15, 35, 38]
	Preparedness and willingness of dental care providers	[24]
	Communication skills	[42]
	Complexity of the child’s medical condition	[43]
	Oral hygiene challenges	[43]
	Inadequately motivation	[35]
	Fear of the dentist and health care providers	[44]
**Access** **barriers**	Challenging behaviours	[25, 32, 43, 45]
	Inadequate insurance coverage	[8, 25, 46, 47]
	Low demand from parents	[48]
	Community disagreement regarding fluoride	[48]
	Social-economic status and lower-income	[9, 49–51]
	Dissatisfaction with dental treatments	[44]
	Costs and financial burden	[49]
	Familial and cultural influences on oral care habits	[49]
	Location and equipment of the dental offices	[41]
	Physical barriers inaccessibility to a dental office	[41]
	Limited agency resources	[49]
	Lack of training program for undergraduate and Curricular changes	[44, 52]
	Affordability	[8, 43]
**Policy** **advice**	Engaging non-dental providers	[53]
	Restrictive administrative and system-level policies	[53]
	Coordination between community-based organizations, health providers, and advocates	[54, 55]
	Increased experience for general dentists through residency training	[44]
	Improving special care dentistry training	[44, 54]
	Increasing the number of general dentists	[54]
	Dental community in coordination with early intervention providers.	[55]
	Engagement of multidisciplinary professionals in CSHCN’s checkups	[56]
	Proposing health-care delivery services that increase coordination and access	[57]
	Development of effective oral health programs	[30, 57]
**Oral health improvement interventions**	Training to brush the teeth with the help of pictures	[32, 44, 58]
	Basic training of dental professionals in sign language	[44]
	Additional training and education dentist	[40, 44, 59]
	The increasing role of teacher towards dental health care children with special needs	[60, 61]
	Community-based and preventive interventions	[36, 62, 63]
	Improve all caregivers’ oral hygiene skills	[63]57
	Incorporate oral health education into nursing curricula	[59]

### Providers’ perception

Providers’ perception toward service providing for CSHCN includes nine sub-themes. This theme is really important and easily can influence the quantity and quality of the service provision to CSHCN. The providers` negative feelings and perception [40], along with their inadequate knowledge and experience in communicating with these children [41], can affect the access and tendency of the CSHCN and their families to utilize dental services. Furthermore, other emotional reactions by the providers, the same as fear [44] or lack of motivation [35] can intensify this problem.

In this regard, according to DeLucia et al. [24] and Adyanthaya et al. [41], the dentistry students with more experience of working with children with special needs, had a greater tendency and satisfaction of providing services to CSHCN. Improving the educational curriculums and changing their direction toward experimental learning of these patients were among those recommendations that would affect the dentists’ perception [44]. At the same time, more comprehensive knowledge of dentists may cause better perception. So, focusing on training and empowering dentists and their teams with the special skills and knowledge of working with CSHCN can improve their perception and performance [29].

### Intervention for oral health improvement

This theme includes seven sub-themes that all present effective interventions in a wide range of those interventions with the aim of empowering the children with special health care needs, their parents and caregivers and those interventions with the target of enabling dentists, nurses, paediatricians and other healthcare providers to provide more effective services or offer more timeliness referral for dental cares.

Different studies have pointed to various interventions in this regard. For instance, integrating oral and dental health education in the nursing curriculum was among one of the interventions for reaching a better oral in children with special health needs [52]. According to the evidence, improving the attitudes of dentists and other care providers lead to an effective understanding of CSHCN’s needs and consequently hone the quality of the services [59]. Another suggested intervention was training and enabling the dental service providers in the scope of using sign language, particularly for working among hearing-impaired patients [60]. Other interventions proposed a reimbursement system providing additional payments to those practitioners who work with CSHCN [15, 43]. Such interventions, along with those suggested creating a calm and peaceful environment in dental clinics for decreasing the sense of fear and anxiety [44] of the children with special needs, may lead to improving their oral health status.

### Need assessment

Need assessment, as the other theme of provision of dental services in children with special needs, includes eight sub-themes. Summarizing and synthesizing all, this theme shows the different and complex needs of CSHCN in the area of oral and dental services. Although some of their needs are obvious and evidence-based, the same as their poorer status of oral hygiene [9] and their physical or intellectual problems in performing tooth brushing and other routine care [31], the others may be considered as unmet needs and should be regarded by the oral health policymakers as well. Preventive dental services are among the latter one. These kinds of services are more considerable compared the specialized treatments. Thus regarding the preference of preventive dental health services, the need assessment should pay attention to these needs and the other risk factors together with poverty and restrictions related to disabilities in CSHCN [8]. Medical unmet needs can also be considered as an intensive predictor of unmet dentistry needs [49]. So it can be used as an indicator for population need assessment, and the later policies and plans can be implicated based on this. At the same time, oral health promotional behaviours such as routine dental visits and tooth brushing can have a strong relationship with the status of decayed teeth in CSHCN [61].

### Policy advice

Policy advice consists of ten sub-themes. This advice and directions are mainly to try to increase the intra-sectoral coordination as well as multidiciplinary team-based activities and improve the community-based service delivery based on the children’s needs. In this regard, one of the main effective policies is health care providers’ collaboration. This policy can significantly improve the access of children with special needs to dental services [53]. At the same time, policies which aimed at the provision of comprehensive preventive and treatment services for CSHCN can be much more effective than those concentrated only on one separate service [49]. It would be clear that policies directed to reducing the barriers of access to services for CSHCN can be much effective [53].

### Access barriers

Access barriers consist of 13 sub-themes in three classifications of geographical and physical access, cultural and behavioural access and finally the financial access. Among the factors related to financial access, inappropriate insurance coverage is considered as one of the main barriers that can greatly affect the CSHCN’s access. In this regard, evidence confirms that insured children with special needs have a better status of oral health along with having fewer problems in dental visits and fewer unmet needs [46, 47]. Another access barrier is the socioeconomic status of families with children with special health needs. This can lead to poor oral health in CSHCN [49, 53]. More than economic barriers, other restrictions in resources, administrative limitations and referral system problems along with high costs of the services also can affect the families’ demand for dental services [44, 48]. Among other considerable barriers, we can point to the lack of eager and skilful dentists in some regions for working with CSHCN [51, 54].

**Fig. 5:**
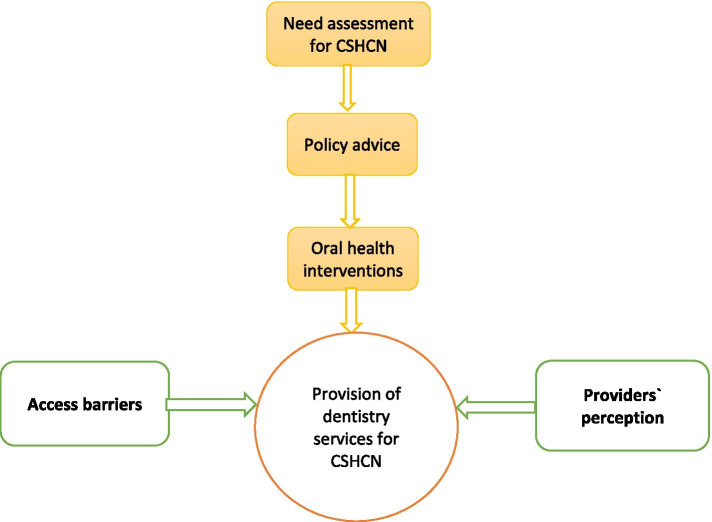
The conceptual map achieved from the scoping review

Finally, at the last step of the scoping review, a mini expert panel was conducted, including two of the researchers (PB and AG) and three other experts in the fields of health policymaking and oral health. Via this mini panel session, the pre-stated dimensions that affected the dentistry services’ provision for children with special needs were presented, and open discussions were conducted about the main themes and sub-themes, and their relations and the final conceptual map of the study was formed. According to this developed conceptual map, oral and dental health need assessment of CSHCN’s services is considered as a starting and fundamental point. According to this need assessment containing both the obvious and declared needs and the unmet needs of CSHCN, the policymakers can set new agendas for policymaking, and in this regard, new policy advice can be formulated. According to these policies, appropriate oral health interventions can be presented. These interventions can be targeted at the CSHCN, their parents or caregivers or the dentists, nurses, paediatricians and other healthcare providers. At the same time, the perceptions of the providers, including their motivations, fears, experiences, knowledge and feeling toward providing services to the CSHCN, can greatly affect the quantity and quality of the provision of oral and dental services to CSHCN. More than these determinants, the geographical and physical access to the provided services along with the cultural and behavioural access and, of course, the financial access to the service that is provided for CSHCN can intensively affect the utilization of these services and the children’s oral and dental health (Fig. 5).

## Discussion

Findings show that the critical oral health need assessment of the children with special needs can be considered as a start point of the conceptual map of providing dental services. This need assessment should distinctly cover a vast range of oral health services, from the unmet needs of the children to their preventive and treatment needs. Oral hygiene improvement is an area of consideration in this regard. Alkhabuli et al. (2019) have emphasized that oral hygiene among Emiratis children with special needs was recorded as good, fair, or poor according to the Simplified Oral Hygiene Index [60]. Another important requirement is educating CSHCN’s parents about their children oral health needs, which can widely affect the reduction of unmet needs [31]. So, considering different perspectives to achieve a comprehensive need assessment can help policymakers move toward more applicable plans and valuable interventions.

According to the present results, review findings, need assessment is an important input for policy advice. Evidence shows that those countries that suffer apparent and need-based policies in the area of oral and dental health, specialy for the high-risk groups can not achieve the oral health goals for the whole community in general and the high-risk groups in a particular perspective [64]. Ghanbarzadegan et al., (2021) have mentioned policy formulation and policy implementation as two determinants of inequality in the provision of dental services. It means that lack of evidence-informed policies based on a comprehensive need assessment can lead to inappropriate policymaking and inequality [65]. For this purpose, these policies should be integrated the assessed needs to the provision of services via making collaboration with health care providers. In other words, dental services can lead to better oral health in children with special health care needs only if policymakers move toward an integrated health care system focused on preventive oral care. According to Hashmi et al. (2019), receiving preventive oral health among CSHCN was reported low. In particular, children registered in managed care programs that had preventive cares coverage also had a better oral health condition compared to those unregistered [60]. This evidence can clarify the role of policies such as health services integration in CSHCN. Other policies which aim for the access of CSHCN to dental services can be considered as well. These policies vary from increasing the CSHCN’s insurance coverage to increasing geographical access to the dental clinics and also improving the cultural access, for instance, improving the children and their parents/carers’ health literacy. So, it is critical for the policymakers to formulate and implement applied policies according to their local condition with the aim of increasing the CSHCN’s oral health access and utilization.

According to the current map, the policy advice based on the primary need assessment have led to appropriate interventions to improve oral health among CSHCN. In this regard, health care systems can implement appropriate and effective interventions according to their resources, social and cultural context. For instance, Krishnan et al., (2019) suggested some ergonomic and psychological interventions such as allocating separated soundproof rooms, playing relaxing music and decreasing the dental instruments’ extra noises in clinics for CSHCN [44]. Other interventions for decreasing the sense of fear or anxiety of CSHCN can be reached according to the health system’s facilities and resources. Other interventions can focus on preventive and oral hygiene practices such as tooth brushing training by using educational videos or posters and with the assistance of trained teachers and social media. Considering the mutual relationship among the CHSCN’s needs, the evidence-informed policies, and the applied interventions is a critical and significant issue for the policymakers. In this regard, Bastani et al. (2021) have also emphasized that lack of effective and practical interventions, policies and practices can be considered as a challenge for the provision and utilization of oral health services [66].

According to the present results, these interventions based on policies advice and previous need assessment can affect the provision of dental services positively in CSHCN. At the same time, providers’ perceptions can widely influence service provision. Adyanthaya et al., (2017) claimed that the most significant barriers as perceived by the practitioners were their level of training and motivation [41]. The importance of perception will intensify knowing that the oral health practitioners’ knowledge, attitudes and perceptions can greatly affect their practices and performances. Altman et al. (2018) claimed that negative perception of health care providers associated with lack of adequate capacity and skills could cause difficulties in service utilization in CSHCN [67]. Another aspect of the importance of providers’ perception in providing care to CSHCN is the comprehensive interaction of CSHCN, their families and the health care system with the health care providers’ capacities and perceptions. So it is recommended to reinforce dental practitioners’ practical skills and positive attitudes toward working for CSHCN.

Finally, the present results indicate that although the need assessment, policies advice, oral health interventions, and the providers’ perception can highly affect the provision of dental services to CSHCN, barriers to access these services can directly restrict the provision process and outcomes. These barriers in any kind of geographical, social, cultural or financial can restrict the children and their families access and affect the provision of oral health services in its intermediate or final indexes such as the DMFT index. Bahadori et al., (2013) emphasized that the cost of services, inconvenience, fear, organization, and patient-dentist relationship are counted as barriers to access to dental services [68]. At the same time, the World’s report on disability (2011) has divided access barriers into health cares in three main dimensions of financial and affordability, barriers to service delivery and human resources barriers [69]. All these access barriers can widely influence the receiving of routine services like dental visits. It is obvious that any change or reduction in access and utilization of dental services by CSHCN can threaten their oral health status as well hamper the performance of the health care system in service provision.

According to what was discussed, the present framework could be applied in different settings. A comprehensive and multi-perspective approach of need assessment can be applied as a basis for evidence-informed policymaking. For this porpuse, both determined and unmet needs of the CSHCN should be considered. Such policies can be accompanied by practical interventions and practices that cover the aimed group’s needs of oral and dental health. It would be obvious that considering different aspects of access to oral and dental health services such as cultural, geographical and financial access can facilitate and develop provision and utilization of the services by all the population as well as the CSHCN. As similarly Ghanbarzadegan et al., (2022) have pointed to the acceptability of oral and dental services by the population and physical and financial aspects as the main determinants of access to dental health [70]. Thus, it can be concluded that for developing such a framework, the policymakers not should pay sufficient attention to the CSHCN’s needs and formulate the policies and design the interventions according to their special needs but also, they should thouroghly consider both the providers` perception and the aspects of access to the services by the consumers.

### Limitations

Although the scoping reviews give a comprehensive conceptual map on the effective determinants on a specific scope, the result of this study can be enriched by applying face to face interviews with some of the children with special health care needs, their parents/carers, oral health practitioners and the health care providers to achieve a broad view. Another limitation of the study can be related to including only published papers in English.

### Strengths

The main strength of the study is presenting not only the main and sub-dimensions of dentistry services` provision for children with special health care needs but also trying to illustrate the relations among these dimensions through a conceptual map. Such a map can shed the light for policymakers for better provision of dentistry services for this vulnerable group. To the best of our knowledge, such a comprehensive scoping review that can shed the light for policymakers to identify the main determinants and design the applied intervention has not been presented before.

## Conclusion

The present findings illustrate a conceptual map of the provision of dental services in children with special health care needs. According to this map, an effective need assessment from this community, including the children and their parents/carers may lead to evidence-informed policymaking and applicable policy advice according to the needs. Such a comprehensive need assessment can be useful in setting new agendas of policymaking or opening the windows in formulating or implementing new policies to improve dentistry services` provision for CSHCN. Then, the policymakers and oral health providers should seek suitable interventions to improve the community’s health literacy, as well as support behaviours of seeking appropriate services by CSHCN. Such interventions can fade the unmet dentistry needs of these children and finally achieve a better status of their oral health. It is also achieved by the results that the policymakers should pay enough attention to restricting the barriers of access to dental services in children with special health care needs. Developing insurance coverage as well as decreasing financial, geographical and social barriers are among some strategies for improving more access to dentistry services by the CSHCN and reducing inequality and disparities.

## Supplementary Information

**Additional file 1.**

## Data Availability

The datasets used and/or analyzed during the current study are available from the corresponding author on reasonable request.

## Abbreviations

CSHCN: Children with special health care needs
SHCN: Special health care needs
