# Supplementary material for: Provision of dental services for vulnerable groups: a scoping review on children with special health care needs
Source: BMC Health Serv Res. 2021 Dec 4;21:1302. doi: 10.1186/s12913-021-07293-4 (PMC8642970; doi:10.1186/s12913-021-07293-4)
Supplement: Supplementary file 1 — Additional file 1. [file 12913_2021_7293_MOESM1_ESM.docx]

**Supplements**

**Table A- The search strategy of the scoping review**

| PUBMED | (((("dental care"[Title/Abstract]) OR ("oral health"[Title/Abstract])) OR (dentistry[Title/Abstract])) AND (((((disability[Title/Abstract]) OR ("special health care needs"[Title/Abstract])) OR ("special needs"[Title/Abstract]))) OR (Unmet need[Title/Abstract]))) AND ("Child"[Mesh] OR "Adult Children"[Mesh] OR "Disabled Children"[Mesh]) |
| --- | --- |
| SCOPUS | TITLE-ABS-KEY ( "oral health" )  OR  TITLE-ABS-KEY ( "dental care" )  OR  TITLE-ABS-KEY ( dentistry )  OR  TITLE-ABS-KEY ( "oral hygiene" )  AND  TITLE-ABS-KEY ( "special health care needs" )  OR  TITLE-ABS-KEY ( disability )  OR  TITLE-ABS-KEY ( "special needs" )  OR  TITLE-ABS-KEY ( "Unmet need" )  AND  TITLE-ABS-KEY ( children )  OR  TITLE-ABS-KEY ( child )  OR  TITLE-ABS-KEY ( child ) |
| ISI Web of Science | #1  **TOPIC:**  ("dental care") *OR* **TOPIC:**  ("oral health") *OR* **TOPIC:**  (dentistry) *OR* **TOPIC:**  ("oral hygiene")  #2  **TOPIC:**  (disability) *OR* **TOPIC:**  ("special health care needs") *OR* **TOPIC:**  ("special need") *OR* **TOPIC:**  ("Unmet need")  #3  **TOPIC:**  (Child) *OR* **TOPIC:**  (children) *OR* **TOPIC:**  ("Adult Children") *OR* **TOPIC:**  (kid)  #3 AND #2 AND #1 |
| PROQUEST | Set#: S2  Searched for: ab("Dental Care") OR ab("Dental Care for Disabled") OR ab("Dentistry") OR ab("Dental Care for Children") OR ab("Oral Health")  Set#: S3  Searched for: ab(Disability) OR ab("special health care needs") OR ab("special needs") OR ab("unmet need")  Set#: S4  Searched for: ab(child) OR ab(kid) OR ab(Children)  Set#: S5  Searched for: S2 AND S3 AND S4 |
| EMBASE | #4. #1 AND #2 AND #3  #3. child:ab,ti OR 'adult child':ab,ti OR 'disabled  children':ab,ti  #2. disability:ab,ti OR 'special health care  needs':ab,ti OR 'special needs':ab,ti OR 'unmet  need':ab,ti  #1. 'dental care':ab,ti OR 'oral health care':ab,  OR dentistry:ab,ti |

**Table B: Summary of the characteristics of included studies**

|  | First Author (Year) | Country | Aim of the Study | Study Design | Sample size and sample characteristics |
| --- | --- | --- | --- | --- | --- |
| 1 | Romer Ocanto (2020) | USA | Describe the training program and its associated evaluation plan | Mix method study | 36 participant |
| 2 | Valendriyani Ningru(2020) | Indonesia | Assess the clinical oral health status and behaviors and treatment needs of people | Cross-sectional | 65 participants with ID |
| 3 | Ashley M. Kranz (2019) | USA | Examin the impact of state policies allowing delivery of POHS in medical offices among Medicaid enrollees | Quantitative study: secondary data analysis | used 2006 through 2014 Medicaid data for children aged 6 months through 5 years . |
| 4 | Ni Zhou (2019) | Hong Kong | Investigate the oral health status and associated factors among CSHCN | cross‐sectional study. | 383 children at Special Child Care Centers |
| 5 | Supriya A (2019) | India | Assessment of Oral Health Status and comparative evaluation of oral Health education methods in CSHCN | non-randomized intervention study | 510 children with special health care needs |
| 6 | Lucía I. Floríndez (2019) | USA | Oral care experiences of latino parents/caregivers with children with autism and with typically developing children | mixed-methods study | 32 Latino parents/caregivers |
| 7 | Isabelle Descamps (2019) | Belgium | Research on the opinions of dentists on the oral health care of children with DS | Quantitative study; cross sectional | 356 dentist |
| 8 | Maureen H. Craig(2018) | USA | Assess preventive dental care use for Medicaid-enrolled CSHCN to Baby and Child Dentistry (ABCD) Program. | cross-sectional | 206488 children |
| 9 | Chairanna, I. (2019) | Indonesia | Understand the effect of increasing role of teachers towards dental health care of CSHCN. | Qualitative study; a case study design | A purposive sample of senior management and public health professionals |
| 10 | Lakshmi Krishnan (2019) | India | Assess the barriers faced by children with disability | Mixed method design: Focus group discussions and in-depth interviews | 195 dentists and 100 caregivers |
| 11 | Gadiyar, A. (2018) | India | Evaluate the relationship between oral health behavior and dental caries experience among CSHCN | Cross-sectional study | 223 CSHCNs from special educational schools |
| 12 | Faisal Saad Al-Harbi (2018) | Saudi Arabia | Examine the relationship between dentist knowledge toward disability and the quality of dental care provided to disabled patients. | Cross-sectional study | 100 dentists and 60 students |
| 13 | H Barry Waldman(2010) | Saudi Arabia | Dentistry for individuals with special needs in Saudi Arabia: a commentary | Commentary | - |
| 14 | Lisa M Delucia (2009) | USA | Relationship between didactic instruction related to CSHCN and dental students' expectations | Quantitative: survey | eighty-two third-year dental students |
| 15 | Nancy Vertel (2017) | Canada | Explore issues of access to dental services for CSHCN | Mix method | 50 caregivers |
| 16 | Sarkar, M. (2017) | Canada | Explores comparative differentials in health care needs, health care utilization, and health | Quantitative: survey | approximately 23 000 Ohio residents between May 2012 and August 2012 |
| 17 | [Amith Adyanthaya](http://www.jisppd.com/searchresult.asp?search=&author=Amith+Adyanthaya&journal=Y&but_search=Search&entries=10&pg=1&s=0) (2017) | India | Investigate the perception of dental practitioners about providing dental care to CSHCN | cross-sectional study | 149 randomly selected general practitioners |
| 18 | Stephanie Cruz (2016) | USA | Identify the types of oral health services offered by community-based organizations to CSHCN and the barriers and facilitators. | Qualitative study, interview | 12 employing key informant interviews |
| 19 | Joseph S. Zickafoose (2015) | USA | Assess how the Children’s Health Insurance Program (CHIP) affects outcomes for CSHCN. | Quantitative: survey | 4142 recent enrollees and 5518 established enrollees responded to the survey |
| 20 | Julie J. Williams (2015) | USA | Barriers to dental care access for CSHCN in an affluent metropolitan community | Quantitative: survey | 385 students at six special education schools |
| 21 | Johanna Norderyd(2015) | Sweden | Describe under 16 year-old children with disabilities receiving pediatric specialist dental care | Mix method | 101 children |
| 22 | Colleen E. Huebner(2015) | USA | Eexamin the preventive dental health care experiences of CSHCN and determine the feasibility of clinical dental examinations | Qualitative: interview | 90 parent interviews |
| 23 | Amitha M. Hegde (2015) | India | Assess the views, attitudes and perceptions of oral health and treatment needs among the parents of 250 children with disabilities | Quantitative: Questionnaire method | 250 parent of children with special need |
| 24 | Carrigan L. Parish (2014) | USA | Examine the oral health knowledge and practices of pediatric nurses coordinating healthcare services for CSHCN | Cross-sectional study | More than 500 nurses in the capacity of either care coordinators or nursing supervisors |
| 25 | Maria Campanaro (2014) | USA | Examine the oral hygiene routines of CSHCN to identify caregiver behaviors and beliefs. | Qualitative: interview | Ninety caregivers of CSHCN |
| 26 | Priscilla Soares Salles (2012) | Brazil | Investigate the dental needs and management of CSHCN | Secondary data analysis | Records of 428 0- to 19-year-old patients |
| 27 | Waldman HB (2011) | Mexico | Dentistry for Mexicans with Special Needs | Commentary | - |
| 28 | Meggan Krause (2010) | United States and Canada | Explore how U.S. and Canadian dental schools educate students about CSHCN | web-based survey | twenty-two dental schools in the United States and Canada |
| 29 | Hiroko Iida (2010) | USA | Dental care needs, use and expenditures among U.S. children with and without  SHCN | Quantitative: Panel Survey | 2005 Household  Component (HC) of MEPS |
| 30 | H. Barry Waldman (2010) | USA | Hardships of raising CSHCN | Commentary | - |
| 31 | Glassman, P (2009) | USA | Planning Dental Treatment for People with SHCN | View point | - |
| 32 | Heather Beil (2009) | USA | Dental care utilization and expenditures in CSHCN | Medical Expenditures  Panel Survey | 8,518 children aged 2 to 17 years |
| 33 | Jean M. Mitchell  (2008) | Columbia | Receipt of preventive dental care among CSHCN enrolled in Medicaid | Secondary data analysis | enrolment and claims records for children with SHCN |
| 34 | Debra Kane  (2008) | USA | Examined the relationship between receipt of routine medical care and receipt of dental care among CSHCN | Cross-sectional study | 2089 patient with special need |
| 35 | Martine Hennequin (2008) | France | Determine inequalities in oral health for children with disabilities | Cross-sectional study | 2487 children with disability, 4772 adolescents and 1641 children without disability |
| 36 | Maryam J Dehaitem (2008) | USA | Explore how dental hygiene programs in the US educate their students about treating CSHCN. | cross-sectional web-based survey | 102 U.S. dental hygiene programs |
| 37 | Fouad S. Salama  (2019) | Saudi Arabia | Evaluate awareness and knowledge of dental students concerning oral-health care for persons | Cross-sectional | evaluated a sample of (150/30) from each of the five dental levels at College of Dentistry |
| 38 | Folakemi A Oredugba (2006) | Nigeria. | Determine the use of oral healthcare services by CSHCN | cross-sectional study | 125 parents of children |
| 39 | Charlotte Lewis (2005) | Columbia | Describe the magnitude of unmet needs for dental care among CSHCN | cross-sectional study | 750 CSHCN from each of the 50 states and the District of Columbia. |
| 40 | Ronald L. Ettinger (2004) | US, Britain, Australia, and New Zealand | Advocate the development of a career path within the field of Special Needs Dentistry | Editorial | - |
| 41 | DANIA E. AL AGILI (2004) | USA | Determine the perceptions of access and barriers to dental care for their children. | Cross-sectional study | 714 parents of children aged 3 to 13 years with special needs |
| 42 | Constance Brooks (2002) | Columbia | Program evaluation of mobile dental services for CSHCN | Cross-sectional study quantitative  survey | 645 parents and  caretakers who received mailed surveys |
| 43 | Paul W. Newacheck (2000) | USA | Assess the role health insurance in influencing access to care and use of services by CSHCN | National Health Interview Survey on Disability | 57 553 children  younger than 18 years old |
| 44 | Donald L. Chi (2013) | USA | Develop a comprehensive family-centred interventions and policies to improve access to preventive dental care for CSHCN. | Questionnaire survey | CSHCN ages 3–17 years with complete  (N = 33904) |
| 45 | Camoin A(2020) | France | Examine the dentists preferences and therapeutic choices for CSHCN. | Questionnaire survey | 139 practitioners |
| 46 | Cleave JV (2008) | USA | To compare dental visits for CSHCN and children without special health care needs | National survey | 102353 children aged under 17 years |
| 47 | Lewis C (2005) | Colombia | Describe the unmet needs for dental care among CSHCN | National survey | 750 CSHCN |
| 48 | Newacheck PW (2005) | USA | Compare utilization and expenditure patterns among CSHCN and those without special health care needs | Panel survey | 6965 children under 18 years old |
| 49 | Song PH (2020) | USA | Examine changes in dental service`s patterns of use by Medicaid-eligible CSHCN | Before-After study | Children with special health care needs |
| 50 | Altaf H Shah (2020) | Saudi Arabia | Effectiveness of Preventive Oral Health Measures among Special Care School Children (boys) in Al-Kharj, Saudi Arabia | A longitudinal study | 163 SCSC (boys) from eight primary schools |
| 51 | Jeffrey N. Lee (2020) | USA | Oral health behaviours and dental caries in low-income children  with special healthcare needs: A prospective observational study | prospective observational study | 116 CSHCN ages 7-20 years from Medicaid enrolment files in Washington state, |
| 52 | Szu-Yu Hsiao (2021) | Taiwan | Dental Treatment Needs and Related Risk Factors among School Children with Special Needs in Taiwan | cross-sectional study | 484 children, aged from 6 to 12 years old |
| 53 | Ashima Goyal (2020) | India | Awareness among pediatricians regarding oral health care in children including those with special health care needs: A cross‑ sectional survey | cross‑ sectional survey | 102 young paediatricians pursuing their Residency Program at the center, working as either Junior Residents or Senior Residents. |
| 54 | Ayako Ide-Okochi (2021) | Japan | Pediatric dentists’ perspectives of children with special health care needs in Japan: developmental disabilities, phobia, maltreatment, and multidisciplinary collaboration | Qualitative research | 21 pediatric dentists |
| 55 | Yazan Hassona (2020) | Jordan | Failure on all fronts: Qualitative analysis of the oral health care experience in individuals with intellectual disability | Qualitative research | Twenty-six parents of 26 individuals with intellectual disability |
| 56 | Paula H. Song (2020) | USA | The effect of an accountable care organization on dental care for children with disabilities | Before-After study | Ohio Medicaid administrative claims data for  year 2011–2016 |
